# Progressive genome-wide introgression in agricultural *Campylobacter coli*

**DOI:** 10.1111/mec.12162

**Published:** 2012-12-20

**Authors:** Samuel K Sheppard, Xavier Didelot, Keith A Jolley, Aaron E Darling, Ben Pascoe, Guillaume Meric, David J Kelly, Alison Cody, Frances M Colles, Norval J C Strachan, Iain D Ogden, Ken Forbes, Nigel P French, Philip Carter, William G Miller, Noel D McCarthy, Robert Owen, Eva Litrup, Michael Egholm, Jason P Affourtit, Stephen D Bentley, Julian Parkhill, Martin C J Maiden, Daniel Falush

**Affiliations:** * Department of Zoology, The Tinbergen Building, University of OxfordSouth Parks Road, Oxford, OX1 3PS, UK; † Institute of Life Science, College of Medicine, Swansea UniversitySwansea, SA2 8PP, UK; ‡ Department of Infectious Disease Epidemiology, Imperial College LondonNorfolk Place, London, W2 1PG, UK; § 451 Health Sciences Drive, 5315, Davis, CA, 95616, USA; ¶ Department of Molecular Biology and Biotechnology, University of SheffieldFirth Court, Western Bank, Sheffield, S10 2TN, UK; ** School of Medicine and Dentistry, University of AberdeenForesterhill, Aberdeen, AB25 2ZD, UK; †† School of Biological Sciences, University of AberdeenForesterhill, Aberdeen, AB25 2ZD, UK; ‡‡ Hopkirk Research Institute, Institute of Veterinary, Animal and Biomedical Science, Massey UniversityPalmerston North, New Zealand; §§ Institute for Environmental Science and Research34 Kenepuru Drive, Porirua, Wellington, New Zealand; ¶¶ United States Department of Agriculture800 Buchanan street, Albany, CA, 94710, USA; *** Health Protection Agency's Centre for Infections61 Colindale Avenue, London, NW9 5EQ, UK; ††† Statens Serum InstitutArtillerivej 5, 2300, Copenhagen S, Denmark; ‡‡‡ Pall Corporation25 Harbor Park Drive, Port Washington, NY, 11050, USA; §§§ Ion Torrent246 Goose Lane, Guilford, CT, 06437, USA; ¶¶¶ Wellcome Turst Sanger InstituteWellcome Trust Genome Campus, Hinxton, Cambridge, CB10 1SA, UK; **** 29 Glebe Road, London, SW130DZ, UK

**Keywords:** adaptation, *Campylobacter*, epistasis, genomics, introgression

## Abstract

Hybridization between distantly related organisms can facilitate rapid adaptation to novel environments, but is potentially constrained by epistatic fitness interactions among cell components. The zoonotic pathogens *Campylobacter coli* and *C. jejuni* differ from each other by around 15% at the nucleotide level, corresponding to an average of nearly 40 amino acids per protein-coding gene. Using whole genome sequencing, we show that a single *C. coli* lineage, which has successfully colonized an agricultural niche, has been progressively accumulating *C. jejuni* DNA. Members of this lineage belong to two groups, the ST-828 and ST-1150 clonal complexes. The ST-1150 complex is less frequently isolated and has undergone a substantially greater amount of introgression leading to replacement of up to 23% of the *C. coli* core genome as well as import of novel DNA. By contrast, the more commonly isolated ST-828 complex bacteria have 10–11% introgressed DNA, and *C. jejuni* and nonagricultural *C. coli* lineages each have <2%. Thus, the *C. coli* that colonize agriculture, and consequently cause most human disease, have hybrid origin, but this cross-species exchange has so far not had a substantial impact on the gene pools of either *C. jejuni* or nonagricultural *C. coli*. These findings also indicate remarkable interchangeability of basic cellular machinery after a prolonged period of independent evolution.

## Introduction

Bacterial genomes show great flexibility in their genome size and composition and can acquire genes encoding entire metabolic pathways from other organisms (Lawrence 1999; Ochman *et al*. 2000). Nevertheless, there are many species that are characterized by a large and stable ‘core genome’. Although DNA within the core genome can be replaced in recombination events, this is almost always with homologous DNA from another member of the same species (Fraser *et al*. 2007). An important evolutionary question is what underlies this stability (Doolittle & Zhaxybayeva 2009). Is the core genome in each species a coadapted unit, such that equivalent genes taken from other species will not function properly? Is each gene in the core genome adapted to the specific sets of environments that the species inhabits? Or is acquisition of DNA limited by mechanisms that prevent uptake of DNA from outside the species?

*Campylobacter* are Gram-negative microaerophilic epsilon proteobacteria that inhabit the intestinal tracts of birds (Waldenstrom *et al*. 2002; Sheppard *et al*. 2010a) and other animals (Rosef *et al*. 1983) and have some capacity to survive in the nonenteric environment (Sopwith *et al*. 2008). They have relatively small genomes of approximately 1.6 megabases (Parkhill *et al*. 2000), which limits the diversity of functions available to each organism. However, in common with many other bacteria, *Campylobacter* shows high levels of recombination (Suerbaum *et al*. 2001; Snipen *et al*. 2012), which might compensate for small genome size by providing each organism with the ability to import genes that confer adaptations to specific environments.

*Campylobacter jejuni* and *Campylobacter coli* are among the main causes of human gastroenteritis worldwide, largely because of infection of farm animals and transmission through the food chain to retail products (Sheppard *et al*. 2009). Both species are associated with several agricultural hosts (Sheppard *et al*. 2010a). *C. jejuni* are usually more abundant in cattle and chickens, and *C. coli* dominates in pigs (Thakur *et al*. 2006). *C. jejuni* has also been isolated from many wild bird species (Waldenstrom *et al*. 2007; Colles *et al*. 2008), but little is known about the distribution of *C. coli* among wild hosts.

*Campylobacter*-like organisms were described by Theodor Escherich in 1886 (Escherich 1886), and the genus was formally named in 1963 (Sebald & Veron 1963). *C. coli*, identified as *vibrio* isolated from pig faeces (Doyle 1948), was designated as a species distinct from *C. jejuni* in 1973 (Veron & Chatelain 1973), and this species classification has been uncontroversial. However, by analysing the sequence from 7 housekeeping loci from a large number of strains, we found evidence for the acquisition of substantial amounts of *C. jejuni* DNA by one of the three *C. coli* clades (Sheppard *et al*. 2008, 2011). Both the correctness and implications of this finding have been debated (Cohan & Koeppel 2008; Doolittle 2008; Caro-Quintero *et al*. 2009; Lefebure *et al*. 2010), and many questions remain about the patterns of introgression in the core and pan genome, and how it has influenced the evolution of these important pathogens.

Here, in addition to four previously published genomes, we chose 26 *Campylobacter* isolates from clinical, agricultural and nonagricultural sources (Tables S1 and S2, Supporting information), to encompass the known diversity based on analysis of seven housekeeping loci (Sheppard *et al*. 2009). The genomes of the isolates were sequenced using the Illumina GA and Roche 454 platforms and assembled *de novo*. We performed model-based analysis of evolution of the core and pan genomes to reconstruct a history of between-species genetic exchange within the genus.

## Materials and methods

### Isolates and sequencing

Isolates were chosen from multilocus sequence-typed collections to represent known diversity among *C. jejuni* and the three major *C. coli* clades, including nonagricultural strains for each lineage. These were cultured and genomic DNA was sequenced using Roche GS-FLX or Illumina Genome Analysers (see Appendix S1). Details of all the isolates used, including four complete *C. jejuni* genomes (Parkhill *et al*. 2001; Fouts *et al*. 2005; Pearson *et al*. 2007) from the NCBI database (accession numbers: NC_009839; NC_008787; NC_003912; NC_002163), are included in Tables S1 and S2 (Supporting information).

### Genetic relationships between *C. coli* and *C. jejuni*

A schematic diagram of the genomics analysis pipeline is given in Fig. S1 (Supporting information). The Bacterial Isolate Genome Sequence Database (bigsdb) (Jolley & Maiden 2010) was used to store contiguous sequences and whole genome data from Genbank. Locus names and reference sequences were defined based upon the finished genome of isolate NCTC11168 (Cabello *et al*. 1997; Parkhill *et al*. 2001; Gundogdu *et al*. 2007). The presence of a preliminary set of orthologs was defined by identifying reciprocal best hits to 11 168 loci, with at least 70% nucleotide identity and 50% difference in alignment length using the blast algorithm. The analysis of orthology was made for every genome, and the core genome, consisting of genes ubiquitous among isolates of the genus, was defined.

Gene orthologs were aligned on a gene-by-gene basis using muscle (Edgar 2004) and then concatenated into contiguous sequence for each isolate genome including gaps for missing nucleotides (or entire genes). A phylogeny of whole genome alignments (1.53 Mbp) was reconstructed using mega (Kumar *et al*. 2008) version 3.1 with the Kimura 2-parameter model and neighbour-joining clustering.

The ancestry of individual nucleotides was estimated using the model-based clustering algorithm implemented in the software structure (Falush *et al*. 2003). A file describing all the 239 543 nucleotide substitutions and the position of the polymorphic sites was constructed from the gene-by-gene alignment file, for loci present in all the genomes. structure was run for 100 000 iterations following a 20 000 iteration burn-in. Genes were ordered by the amount of introgression in the 828 complex, identified with structure. For the 13 most introgressed genes, also present in the analysis of Lefebure *et al*. (2010), individual neighbour-joining trees were constructed with and without our strains (Fig. S8, Supporting information).

Pairwise alignments of genomes were generated using progressive mauve version 2.3.1 (Darling *et al*. 2004, 2010) with the default parameter settings and analysed using a Bayesian change point model as described previously (Didelot *et al*. 2007). This model assumes that the level of nucleotide divergence between the two genomes follows a stepwise constant function and uses a reversible-jump MCMC to reconstruct this function. Histograms were then built to show the distribution of the level of divergence along the genomes.

### Event based analysis of *C. coli* clade 1 evolution

Multiple sequence alignment of the contigs for each genome was performed using progressive mauve version 2.3.1 (Darling *et al*. 2004, 2010) with the default parameter settings. The progressive mauve backbone output file was used to assign regions of each genome as either core (‘backbone’) segments, conserved among all of the genomes, or accessory (‘variable’) segments absent from at least one alignment. Briefly, the multiple genome alignment was automatically analysed to identify conserved segments using a homology hidden Markov model (Treangen *et al*. 2009). Regions where the posterior probability of sequence homology was >90% using a model trained on 80% identity and tuned to the sequence composition of *Campylobacter* were considered to be homologous. Nonhomologous regions create alignment gaps, and those alignment gaps were used to delineate a ‘backbone’ of conserved segments among each pair of genomes by simply calling any region with >20 nucleotides inserted or deleted in one genome as nonbackbone (indels > 20 nt). Pairwise backbone predictions were merged into multigenome backbone predictions using the previously described methods (Treangen *et al*. 2009). Using this technique, the amount of core and accessory genome was determined for all the isolates and for *C. jejuni* and *C. coli* individually and the three *C. coli* clades separately and for subsets of isolates (Fig. S9, Supporting information).

Additionally, a gene-by-gene alignment was extracted from bigsdb (Jolley & Maiden 2010) for genes present in all the *C. coli* clade 1 genomes. A genealogy for these alignments was estimated using clonalframe, a model-based approach to determining microevolution in bacteria (Didelot & Falush 2007). This programme differentiates mutation and recombination events on each branch of the tree based on the density of polymorphisms. Clusters of polymorphisms are likely to have arisen from recombination and scattered ones from mutation. Run on the *C. coli* clade 1 alignment, clonalframe, estimated that recombination introduced polymorphism at an average of 8% of affected sites. This value is higher than the genetic diversity within *C. coli* clade 1 and thus corresponds to imports from the other clades and species, as well as back-recombination events replacing previously introgressed DNA. The programme was run with 50 000 burn-in iterations followed by 50 000 sampling iterations. The consensus tree represents combined data from three independent runs with 75% consensus required for inference of relatedness. For each branch on the clonalframe genealogy, a list of homologous recombination events was extracted. Recombination events were defined as sequences of length >50 bp with a probability of recombination ≥75% over the length reaching 95% in at least one site.

To investigate the acquisition and loss of nonhomologous DNA, we used the model-based Bayesian method implemented in genoplast (Didelot *et al*. 2009). This model allows the rates of gain and loss of genetic elements to be investigated over time in individual lineages. A multiple alignment was produced for *C. coli* clade 1 genomes (excluding isolate 16) using progressive MAUVE (Darling *et al*. 2004, 2010), and the conserved orthologous segments and repeat elements defined the core genome. Large gaps (≥500 bp) in the alignment, where one or more genomes contain a sequence absent in the other genomes, identify the position of imported DNA in the accessory genome. A binary matrix of presence/absence of genetic features of length 50 bp was constructed using the bbFilter script distributed with MAUVE. genoplast was run with default parameters using this matrix and the genealogy inferred by clonalframe as input.

The origin of homologous and nonhomologous recombination events in *C. coli* clade 1 genomes was determined using the blast algorithm (Altschul *et al*. 1990). A list of events identified with clonalframe and genoplast was extracted, and sequences were compared to a library database of all the *C. jejuni* and *C. coli* clade 2 and 3 genomes. The origin of the events was assigned based on the similarity (S) to a library sequence. Specifically, the *E-*value that measures the reliability of the S score was calculated for all blast matches (≥70% identity, 50% alignment), and the event was inferred to have originated in the species/clade containing homologous sequence with the lowest *E*-value.

In addition to determining the clonal genealogy and the origin of recombination, clonalframe analysis information was used to investigate the impact of homologous recombination with *C. jejuni* on sequence divergence in *C. coli* clade 1 using sequence variation at 51 ribosomal protein (*rps*) subunit loci (see Appendix S1).

### Functional analysis of introgression

The proportion of nucleotide substitutions that changed the amino acid sequence in homologous sequence was investigated for recombinant *C. jejuni* genes found in *C. coli* clade 1. Genome comparison was made between an example *C. jejuni* (isolate 4) and an unintrogressed *C. coli* (isolate 23). These strains shared 1081 genes, defined as homologous sequence with >70% identity over >50% of the gene alignment; 584 of these genes were involved in recombination in at least one *C. coli* clade 1 isolate and 497 were not. The number of nonsynonymous differences (*N*), number of synonymous differences (S) and ratio of nonsynonymous to synonymous mutations (dn/ds) were determined from gene-by-gene alignments of recombining and nonrecombining genes using mega software version 3.1 (Kumar *et al*. 2008). If recombinant sequence is removed by the action of selection against divergent amino acid sequence, then there will be a greater than expected number of synonymous substitutions in recombined genes and a lower dn/ds ratio.

To investigate the relationship between the rate of genetic import and the functional category of genes, the number of genes involved in homologous recombination was determined for each cluster of orthologous groups (COG) category. For each COG category, the number and total length (bp) of imports were determined. This allowed the determination of the rate of imports per nucleotide and the proportion of genes from each COG involved in recombination. Some genes are present in two or more COG categories and were counted once for each COG. A second analysis of the function of recombined genes was carried out by determining the genes that were found only in *C. jejuni* and *C. coli* clade 1 and were absent in unintrogressed *C. coli*.

Genome comparison was carried out to identify differences in the gene content of the *C. jejuni* and *C. coli* genomes. By organizing *C. jejuni* genes absent from unintrogressed *C. coli* into functional categories, groups of genes of related function were identified. A second comparative analysis identified genes that were found only in *C. jejuni* and introgressed *C. coli* genomes.

## Results

Our initial analysis focussed on the core genome. The NCTC11168 isolate has a 1.6 Mb genome (Parkhill *et al*. 2000), and 0.96 Mb was aligned in all our isolates using mauve. Based on locus designations for NCTC11168 (1623 genes), there were 542 genes with orthologues in all the isolates (genes with at least 70% nucleotide identity and a minimum of 50% alignment length). Under the same criteria, there were 819 genes (50%) common to all *C. jejuni* isolates and 928, 1084 and 1078 common to *C. coli* clades 1, 2 and 3, respectively.

**Fig. 1:**
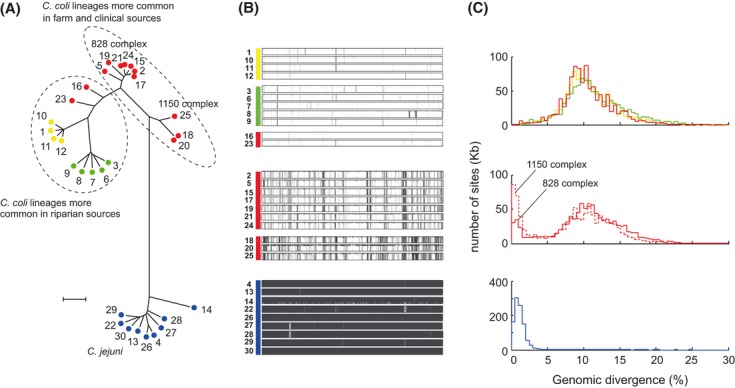
Ancestry of *Campylobacter jejuni* and *C. coli*. (A) Neighbour-joining tree of 30 *C. jejuni* and *C. coli* genomes. Isolates belonging to *C. jejuni* are shown in blue, and those belonging to *C. coli* clade 1 are indicated in red, clade 2 in yellow, and clade 3 in green. The scale bar represents a genetic distance of 0.01. (B) Genetic ancestry of 239 543 polymorphic sites among *C. jejuni* and *C. coli* isolates inferred using structure assuming 2 populations. Genomes are ordered according to isolate NCTC 11168(Parkhill *et al*. 2000), and each nucleotide is coloured according to genetic ancestry to *C. jejuni* (▪) or *C. coli* (□). (C) Histograms of nucleotide divergence between *C. jejuni* (isolate 4) and genomes from *C. coli* clade 1 (red), 2 (yellow), 3 (green) and *C. jejuni* (blue). Pairwise comparisons between *C. jejuni* and un-recombined clade 1 (isolate 23 in the example), and clade 2 and 3 genomes (upper panel) show a unimodal distribution with modes between 10 and 12%. Comparison of *C. jejuni* with *C. coli* clade 1 isolates from the ST-828 and ST1150 complexes (middle panel) has a bimodal distribution with similar modes at 10–12% but with an earlier mode at <2%. The nucleotide divergence of the earlier mode is similar to comparison between two *C. jejuni* genomes (bottom panel).

We first constructed a neighbour-joining tree based on average genetic distances amongst isolates (Fig. 1A). On the tree, *C. jejuni*, *C. coli* clade 2 and *C. coli* clade 3 isolates each formed discrete clusters. However, isolates previously designated as *C. coli* clade 1 were found in three places on the tree. The ST-828 and ST-1150 clonal complexes, which account for the great majority of strains found in agriculture and human disease (Sheppard *et al*. 2010b), formed discrete clusters separate from the two environmental *C. coli* clade 1 isolates.

### Evidence for introgression

Three lines of evidence show that the large genetic distances among *C. coli* clade 1 isolates, illustrated by the neighbour-joining tree (Fig. 1A), are a consequence of the import of DNA from *C. jejuni* rather than accumulation of mutations during a prolonged period of separate evolution. The first used the linkage model of structure (Falush *et al*. 2003) that reconstructs ancestral populations from DNA polymorphism data. When run assuming two ancestral populations, the inferred ancestral sources corresponded to *C. jejuni* and *C. coli*. The human and agricultural *C. jejuni* isolates had between 0.4% and 1.7% inferred *C. coli* ancestry, consistent with a low level of import. Excluding isolates from the ST-828 and ST-1150 clonal complexes, the *C. coli* isolates showed a comparable amount of inferred *C. jejuni* ancestry that ranged from 0.2% to 1.2%. The ST-828 and ST-1150 clonal complexes showed substantially more evidence for DNA import from *C. jejuni* ranging from 9.7% to 11.2% and 20.4% to 22.5%, respectively, spread throughout the genome (Fig. 1B).

The second line of evidence for introgression into *C. coli* clade 1 is provided by pairwise comparison of nucleotide differences between genomes. In the absence of gene flow, isolates from the two species should have a unimodal distribution of divergence levels reflecting accumulation of mutations throughout the genome. This pattern was observed for comparisons between *C. jejuni* and unintrogressed *C. coli* isolates, with modes between 10% and 12% (Fig. 1C). Comparisons with the ST-828 and ST-1150 clonal complex isolates showed a bimodal distribution with similar modes at 10–12% but also earlier modes at <2%. The low nucleotide divergence was consistent with recent recombination with *C. jejuni*. Combined with evidence for shared polymorphism found using structure, these patterns of divergence are consistent with recent gene flow from *C. jejuni*. The imported DNA has greater nucleotide identity to the agricultural *C. jejuni* isolates than the environmental *C. jejuni* isolates (Fig. S2, Supporting information).

**Fig. 2:**
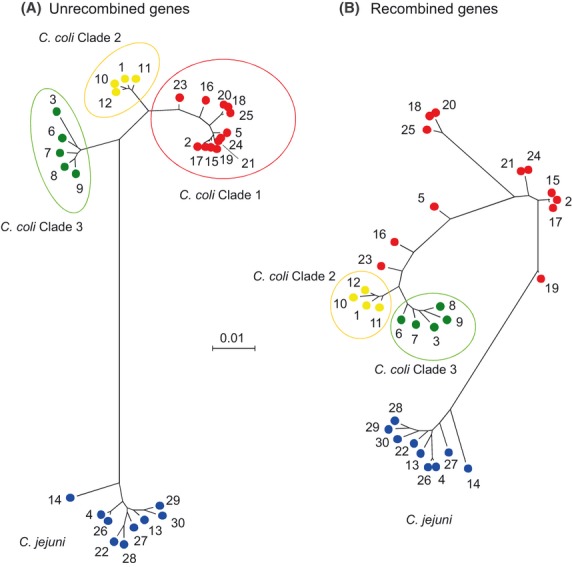
The effect of interspecific recombination on tree clade structure. Maximum likelihood trees, based on the Tamura–Nei model, of 30 *Campylobacter jejuni* and *C. coli* genomes are based on concatenated sequences of ribosomal protein (*rps*) subunit loci genes that show (A) 35 genes with no evidence of homologous recombination and (B) 16 with evidence of recombination in at least one isolate using clonalframe. Isolates belonging to *C. jejuni* are shown in blue, and those belonging to *C. coli* clade 1 are indicated in red, clade 2 in yellow, and clade 3 in green. The trees are drawn to scale, with branch lengths measured in the number of substitutions per site. The scale bar represents a genetic distance of 0.01. Trefoil clade structure is resolved in nonrecombining genes.

The third line of evidence is provided by constructing maximum likelihood trees separately for loci according to whether they have any *C. jejuni* ancestry according to clonalframe. This analysis was performed for the 51 ribosomal protein subunit (*rps*) loci in our genomes (Jolley *et al*. 2012). When analysis was limited to genes where there was no *C. jejuni*-like sequence, the clade 1 strains clustered together (Fig. 2), consistent with their shared common ancestry as was found previously for seven MLST loci (Sheppard *et al*. 2008). Furthermore, as in this previous analyses, the branching pattern positioned clade 2 as a sister taxa to clade 1. In contrast, on the tree for *rps* loci that showed evidence of interspecies recombination, *C. coli* clade 1 isolates were scattered around the branches joining the two species in the tree (Fig. 2). This analysis suggests that *C. coli* clade 1 is a real clade and that the presence of clade 1 isolates on three different parts of the whole genome neighbour joining (Fig. 1A) is an artefact of the substantial effect of interspecies recombination on genetic distances.

### Core and pan genome evolution

**Fig. 3:**
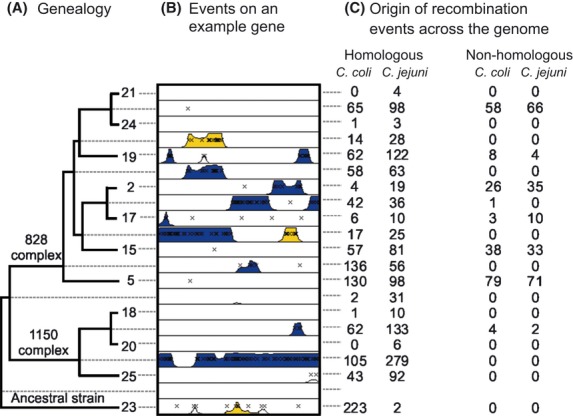
History of recombination in *C. coli* clade 1. (A) Genealogy inferred using clonalframe. (B) Recombination and mutation events on each branch of the genealogy, for an example, 1046 bp gene (metC2). Crosses indicate substitutions by either mutation or recombination. Recombination events inferred with high posterior probability are coloured according to whether the imported DNA is more similar to genomes from *Campylobacter jejuni* (blue) or *C. coli* clades 2 and 3 (yellow). (C) Total number of inferred homologous and nonhomologous imports on each branch from each species across the genome. Isolate 16 was excluded from this analysis because of poor genome coverage.

Having established that up to 23% of the *C. coli* clade 1 genome is of *C. jejuni* origin, we investigated evolution within the clade and the sequence of events responsible for introgression. An alignment of *C. coli* clade 1 genomes was constructed (excluding one strain with low genome coverage) and a tree of clonal relationships was estimated using clonalframe (Didelot & Falush 2007). The analysis showed that the ST-828 and ST-1150 clonal complexes are more closely related to one another than to an environmental isolate (isolate 23) (Fig. 3A). Most of the *C. jejuni* DNA found in the ST-828 complex was also found in the ST-1150 complex (Fig. 1B and Fig. S3, Supporting information), implying that this genetic material was imported by the common ancestor(s) of both complexes. Subsequent to the divergence of the two complexes, the ST-1150 complex has acquired substantially more *C. jejuni* DNA than the ST-828 complex although import is ongoing in both complexes.

In addition to recombination of homologous sequence, our approach allows us to investigate the evolution of the pan genome, which occurs via acquisition and loss of genes. A multiple genome alignment was constructed for *C. coli* clade 1 isolates using mauve (Darling *et al*. 2004), and genoplast (Didelot *et al*. 2009) was applied to the alignment blocks to identify those that were gained or lost on particular branches of the tree (Fig. 3C). The origin of imported DNA was inferred by blast comparison of the sequences to reference genomes from *C. jejuni* and *C. coli* clade 2 and 3. An equivalent analysis of origin was performed for homologous imports (Fig. 3C). In total, 438 nonhomologous and 2237 homologous recombination events were inferred, although for methodological reasons, both homologous and nonhomologous events were only identified reliably towards the tips of the tree. In both cases, approximately 50% of events were of *C. jejuni* origin although for homologous recombination, the proportion is higher for many of the short branches. This demonstrates that a high magnitude of introgression has occurred in both the core and pan genomes.

### Effects of introgression on the core genome

**Fig. 4:**
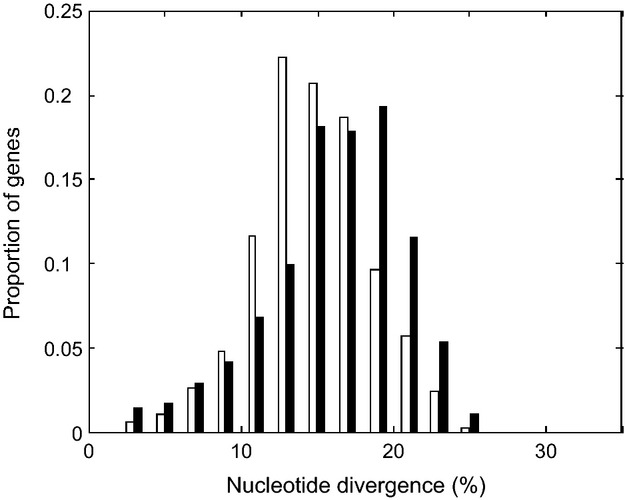
Homology dependence of recombination between *Campylobacter jejuni* and *C. coli*. The distribution of divergence between *C. jejuni* (isolate 4) and unintrogressed *C. coli* clade 1 (isolate 23) for the recombinant genes (white) and the nonrecombinant genes (black). Recombination is rarer in areas of the genome where there is high divergence between species but the effect is slight with a large overlap between the two distributions. Recombination occurs between genes at all levels of divergence.

The availability of unintrogressed *C. coli* genomes provides insight into where introgression has occurred and its genomic effects. Data in a variety of bacterial species have suggested that recombination is homology dependent (Cohan 2002; Fraser *et al*. 2007). We found that recombination was rarer in areas of the genome where there was high divergence between *C. jejuni* and the unintrogressed *C. coli* (Fig. 4). However, the observed degree of homology dependence was several orders of magnitude weaker than in other species where mismatch repair mechanisms prevent the integration of most sequences that contain even small numbers of nucleotide differences (Cohan 2002; Fraser *et al*. 2007). Based on a conservative threshold for identifying interspecies recombination events, clonalframe analysis implied that 9 (95% credibility regions 5–16) times as many substitutions were introduced on average by interspecies recombination as by mutation or intraspecies recombination. Within the most divergent regions of the genome (approximately 20% nucleotide divergence), the rate of interspecies exchange is approximately half the genome-wide average, but this still implies nucleotides are at least four times more likely to be changed by cross-species recombination than by new mutation or within-species recombination. This level of recombination would lead to progressive species convergence if maintained throughout the genome over time (Sheppard *et al*. 2008).

Comparing a unintrogressed *C. coli* clade 1 isolate (isolate 23) with a *C. jejuni* genome (isolate 27) shows that there are on average 38 nonsynonymous substitutions per gene. This is approximately an order of magnitude more than in pairwise comparisons within unintrogressed *Campylobacter* populations, which range from 1.4 for *C. coli* clade 2 to 5.5 for *C. jejuni*, comparable to 2.9 nonsynonymous substitutions per gene between human and chimpanzee, for example (Consortium(TCSaA) (2005). We found that introgression has taken place at equivalent rates in regions of both high and low nonsynonymous differentiations between the species (Fig. S4, Supporting information) and that there are on average 9.8 protein coding differences per gene between an unintrogressed clade 1 isolate (strain 23) and a member of the ST-1150 complex (strain 25). There was also no evidence for large differences in the rate of introgression between broadly defined functional categories (Fig. S5, Supporting information). Thus, introgression has greatly increased overall genetic diversity across the genome in *C. coli* clade 1 and introduced thousands of changes that have potential functional significance.

### Effects of introgression on the accessory genome

Genome comparison identified differences in gene content of *C. jejuni* and *C. coli*. By organizing the 88 *C. jejuni* genes absent from unintrogressed *C. coli* into functional categories, large groups of genes of related function were identified (Table S3, Supporting information). Particularly notable are: (i) solute transporters of different families, which may reflect differential nutrient utilization in the two species; (ii) specific cytochromes *c* and their associated biogenesis proteins, which may be related to the use of specialized respiratory substrates; and (iii) TonB-dependent outer membrane proteins potentially involved in iron uptake. The list also includes genes in diverse functional categories including those involved in core cellular function. Detailed characterization of the functional consequences of these differences requires further investigation.

Thirty-one genes were identified only in *C. jejuni* and introgressed *C. coli* based on blast similarity (Table 1). Several genes were from a region (*Cj0480c*–*Cj0490*) that has recently been shown to be involved in the transport and metabolism of L-fucose (Muraoka & Zhang 2011). Most sugars cannot be used as growth substrates for *Campylobacter*, due to lack of 6-phosphofructokinase (PFK) (Velayudhan & Kelly 2002), so these genes presumably allow conversion of fucose to triose phosphate or another intermediate that bypasses PFK for entry into central metabolic pathways. Fucose is a major component of host glycoproteins, particularly intestinal mucin, and obtaining these genes from *C. jejuni* could allow *C. coli* to utilize fucose and provide an advantage in the gut (Stahl *et al*. 2011). The list of genes present only in *C. jejuni* and introgressed *C. coli* also includes key genes associated with flagella. Phylogenetic trees of the fucose-associated genes show some isolates have sequences with high nucleotide identity to those found in *C. jejuni*, but other sequences are entirely distinct, suggesting that the genes were present in the *C. coli* pan genome prior to introgression and have been lost by unintrogressed strains (Fig. S6, Supporting information). Intriguingly, several distinct genotypes exist among the *C. jejuni*-like sequences consistent either with rapid diversification or with introgression on multiple occasions, suggestive of recent selection at that locus (Falush 2009).

**Table 1:** *Campylobacter jejuni* genes with homologous sequence (70% blast similarity, >50% of the gene) present among introgressed *C. coli* clade 1 genomes and hypotheses about their potential function

Gene	Product	Description*—*Hypothesis
Transport and metabolism of L-fucose		
Cj0480c	Transcriptional regulator	This is divergently transcribed from the other genes in this unit and is likely to be regulating the rightward reading genes. Cj0480 is an IclR family regulator (Gundogdu *et al*. 2007) and could be acting as a repressor or activator inducing expression of catabolic and transport genes in response to L-fucose
Cj0481 (annotated as *dapA*)	Putative dihydropicolinate (DHP) synthase	DHP, also present in *Bacillus* spores, is an intermediate of a variant of the lysine biosynthesis pathway from aspartate that catalyses the condensation of aspartate semi-aldehyde with pyruvate to form DHP. Cj0480 may catalyse a related lyase reaction, as there is another gene Cj0806 that could be the ‘real’ *dapA*; it may be involved as a lyase in a step of fucose catabolism?
Cj0482/0483 (*uxaA*)	Putative altronate or D-galactarate (sugar) hydrolase	Could be a pseudogene because the N-terminus encoded in Cj0482 and the C-terminus in Cj0483 is separated by a stop codon. There are examples where such genes are expressed. Possible fucose hydrolase?
Cj0484	Major facilitator superfamily transport protein	Probably, a substrate-proton symporter to import a substrate driven by the pmf. It has some similarity to phthalate (aromatic) family transporters (Gundogdu *et al*. 2007). However, it is not possible to say what the substrate is likely to be from sequence data
Cj0485	Dehydrogenase/oxidoreductase, FabG family.	This is possibly an alcohol dehydrogenase
Cj0486	Probable L-fucose transporter	This is a sugar transporter of the major facilitator superfamily, with significant similarity to the L-fucose–proton symporter of *E. coli* and other bacteria (Gundogdu *et al*. 2007). This is essential for L-fucose utilization in some *C. jejuni* strains (Muraoka & Zhang 2011)
Cj0489/Cj0490	Putative aldehyde dehydrogenase	Potentially involved in a step of fucose catabolism?
Zinc uptake system		
Cj0263	Zinc transporter ZupT	There may be a connection between the zinc uptake system genes in supplying zinc for the activity of the protease. A number of proteins contain the Cj1589 domain, so it is difficult to predict the function but there may be a zinc connection with Cj0263.
Cj0620	Zinc-dependent protease	
Cj1589	Zinc-dependent hydrolase, possibly a beta-lactamase or glyoxalase II	
Flagellin-associated genes		
Cj1339 (*FlaA*)	Flagellin protein	Flagellin-associated proteins that could be involved in niche colonization. The presence of *AcpP2* and *AcpS* could suggest O-linked glycosylation of flagellin proteins being important.
Cj1338 (*FlaB*)	Flagellin protein	
Cj0548 (*FliD*)	Hook-associated protein	
Cj1299 (*AcpP2*)	Acyl carrier protein for the O-linked glycosylation locus	
Cj1409(*AcpS*)	Holo-acyl carrier protein synthase	
Miscellaneous		
Cj0555	Putative malonate (HOOC.CH2.COOH) transporter	This could be involved in growth on malonate, but this is an uncommon plant-derived carbon source.
Cj1297	Putative component of the efflux system	Speculatively associated with antibiotic efflux.
Cj1365c	Secreted serine protease	Could be associated with breakdown of specific proteins for growth on amino acids
Cj1506c (*CcaA*)	Chemoreceptor for aspartate A	Chemotaxis towards aspartate, as facilitated by CcaA, is involved in the colonization of the intestinal tract (Hartley-Tassell *et al*. 2010).
Cj1051c (*CjeI*)	—	A restriction modification enzyme
Cj1134 (*htrB)*	Lauroyl acyltransferase	Enzyme involved in the biosynthesis of LipidA. This will probably be an essential gene
Cj1414c (*KpsC*)	Part of the capsule locus	Probable capsule polysaccharide modification gene
Cj1187c (*ArsB*)	Arsenical efflux pump	Used for detoxification. Actual substrate cannot be predicted, but Cj1297 may also have a related broad detoxification function
Cj0308c (*BioD*)	Dethiobiotin synthase	Involved in synthesis of the cofactor Biotin.

## Discussion

Extensive recent recombination between species has a number of consequences for the patterns of DNA sequence diversity observed. First, there should be regions of the genome where individuals from the two species have highly similar sequence, reflecting recent common ancestry of DNA that has been imported from one species to the other. Second, patterns of inheritance for some stretches of the genome will be inconsistent with the consensus species tree. Third, recombination may elevate diversity within introgressed populations by introducing substitutions that were fixed during species divergence.

Here, we have sought to systematically investigate genome-wide patterns of relatedness between *C. jejuni* and *C. coli* by searching for each of these types of signal within homologous parts of the genome. First, in pairwise comparison of sequence diversity between *C. jejuni* and different *C. coli* isolates, *C. coli* that dominate in agriculture (ST-828 and ST-1150 complexes) had large fractions of the genome with low divergence from *C. jejuni—*typical of that found between two *C. jejuni* isolates. Importantly, isolates from nonagricultural *C. coli* lineages did not have comparable low divergence regions. Second, we used the linkage model of structure to identify sections of the genome with ancestry in both species. Consistent with the pairwise sequence analysis, there was very little evidence for introgression amongst *C. jejuni* and nonagricultural *C. coli* isolates but a high degree of introgression in agricultural *C. coli*. Third, we observed the diversifying effect of recombination directly by reconstructing clonal relationships and the specific imports that took place during the evolution of the agricultural *C. coli* clonal complexes, using clonalframe. We found large numbers of imports that introduced changes at approximately 12% of sites, consistent with an origin in *C. jejuni*. This finding also implies that introgression is ongoing in both the ST-828 and ST-1150 clonal complexes.

### A scenario of *Campylobacter* evolution

**Fig. 5:**
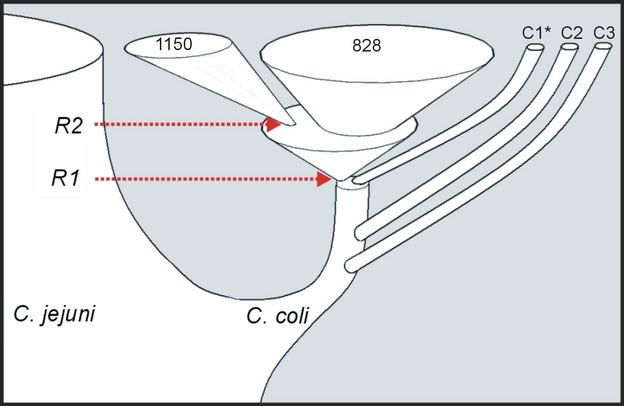
A scenario for the evolution of *Campylobacter jejuni* and *C. coli*. These species diverged followed by the split of *C. coli* clades 1, 2 and 3. Recombination from *C. jejuni* to *C. coli* clade 1 began at some point before R1, and subsequent clonal expansion of introgressed lineages (828 and 1150 clonal complexes) at R1 and R2 led to the dominance of hybrid lineages in agriculture, human disease and currently available isolate collections. Clade 2 (C2) and 3 (C3) and clade 1 (C1*) populations from wild bird and environmental reservoirs (e.g. represented by isolates 16 and 23) remained unintrogressed. The cross-sectional area and diameter of the lineage ‘trunks’ are based on the abundance of isolates in the PubMLST database and the length of trunks is arbitrarily defined.

We have reconstructed an evolutionary scenario (Fig. 5) within which to interpret patterns of diversity in both core and pan genomes. *C. coli* split from *C. jejuni* and subsequently diversified into three clades. Bacteria from both species show evidence of recombination within species and clade (for *C. coli*) from a version of the four gamete test applied to the two most common MLST alleles in each clade population (Sheppard *et al*. 2010c). This has contributed to generating the ‘star-like’ phylogenies amongst the *C. coli* clade 2 and 3 isolates in our sample (Fig. 1). However, genetic exchange between bacteria from different species and lineages has been rare enough to facilitate their progressive divergence, which has reached approximately 12% between *C. jejuni* and *C. coli* and around 4% between the three *C. coli* lineages.

More recently, within *C. coli* clade 1, a lineage arose that started to import substantial quantities of *C. jejuni* DNA. This lineage has given rise to two clonal complexes, the ST-828 and ST-1150 complexes. These two clonal complexes currently make up the great majority of typed agricultural isolates but a small proportion of nonagricultural ones, as shown by the epidemiologically defined sample shown in Table 2. The rate of acquisition of DNA has been substantially higher for the ST-1150 complex than for the ST-828 complex. Because of the great preponderance of agricultural strains in almost all sample collections, several of our *C. coli* clade 2 and 3 isolates that we obtained for genome sequencing come from agriculture (Table S1, Supporting information) but these isolates have not undergone substantial introgression.

**Table 2:** Distribution of *C. coli* lineages among different sources*

		Source					
	
		Farm		Clinical		Riparian	
			
Clade		Isolates	STs	Isolates	STs	Isolates	STs
1	ST-828 complex	915	216	481	86	0	0
1	ST-1150 complex	38	12	0	0	0	0
1	Clade 1 other	201	107	19	17	0	0
2		0	0	0	0	37	31
3		2	2	0	0	30	25

MLST typed farm isolates (from cattle, chicken, pig, sheep or turkey faeces or meat), riparian isolates (from duck, swan, pigeon and gull faeces and environmental water samples),and clinical isolates (from human blood and faeces) are from published studies and defined locations (Sheppard *et al*. 2010b). Most clade 1 isolates that are not part of the ST-828 or 1150 complexes, nevertheless, share alleles with them suggesting recent common ancestry. Note that isolates sequenced for the current study were taken from a wider collection including additional riparian and wild bird isolates.

* Sheppard *et al*. (2010b).

Changes in patterns of gene flow are substantially harder to demonstrate in taxa where divergence is less complete or where there are no unintrogressed ancestral clades preserved. Among bacteria in particular, divergence may be uneven across the genome because of ‘fragmented speciation’ (Retchless & Lawrence 2010). In this model, gene flow ceases first in some parts of the genome, for example, in regions responsible for adaptive divergence. This acts as a barrier to subsequent recombination, and progressive diversification occurs at other loci around the genome. This model is not directly applicable here because isolates found in all three *C. coli* clades had similar high divergence with *C. jejuni* across the genome (Fig. 1C), which implies that after speciation, there was an extended period of divergence with low levels of gene flow. Nevertheless, it is of interest to investigate whether the high rate of genetic differentiation between *C. jejuni* and *C. coli* has acted as a barrier to recent recombination within the two agricultural *C. coli* lineages. Recombination was twice as rare in regions with 20% nucleotide divergence than in regions with 10% (Fig. 4). However, even among divergent regions, the rate of recombination was sufficient to promote progressive species convergence at current levels of DNA exchange.

### Comparison with other studies of introgression in *Campylobacter*

Our results contrast with those of a recent study (Lefebure *et al*. 2010) that analysed a larger number of genomes but failed to find evidence for substantial introgression (except in a single isolate). The disagreement reflects differences in sampling and methodology rather than biology. All but one of the *C. coli* isolates analysed by Lefebure *et al*. (2010) came from the ST-828 complex and were estimated to have <1% introgression on average (Lefebure *et al*. 2010). The single exception was a member of the ST-1150 complex, for which introgression was inferred at 9.6% of genes. We estimated 23% introgression for the same isolate and at least 8% introgression for the ST-828 complex isolates. Moreover, the strains from Lefebure *et al*. (2010) are intermingled with the strains analysed here in a NJ tree constructed using whole genome sequences (Fig. S7, Supporting information).

The substantial underestimation of introgression in the previous study (Lefebure *et al*. 2010) reflects the absence of nonagricultural strains from their sample and the use of a single methodology that is sensitive to sampling. Specifically, Lefebure *et al*. (2010) looked for patterns of ancestry inconsistent with the species tree by using gene-by-gene phylogenies to identify loci where a minority fraction of *C. coli* isolates clustered closer to *C. jejuni* than to other *C. coli*. This approach systematically misses introgression shared by a majority of isolates within the ST-828 complex. Fig. S8 (Supporting information) shows neighbour-joining trees for the 13 genes that showed the highest levels of introgression in our analysis (>70% in each case), constructed with and without the isolates from this study. For most of the genes, the *C. coli* isolates from the study of Lefebure *et al*. (2010) contain sequences similar to those found in *C. jejuni*, while in every case, the nonagricultural *C. coli* isolates in our sample harbour entirely distinct sequences. Even in the cases where a handful of the Lefebure *et al*. (2010) ST-828 complex isolates do have the distinct—and presumably ancestral—*C. coli* sequence, the method they employed did not detect introgression because these isolates make up only a minority of their isolates. As a result, they inferred no introgression for these 15 genes, which is clearly an incorrect conclusion based on visual inspection of the neighbour-joining trees constructed using the combined sample (Fig. S8, Supporting information).

A further limitation of the method employed by Lefebure *et al*. (2010) is that because phylogenies were constructed only for entire genes, they were likely to miss imports of short gene fragments. The linkage model of structure uses a hidden Markov model, which can detect any tract long enough to introduce polymorphisms at several sites that are characteristic of the other species, and we observed many such imports that were much shorter than entire genes (Fig. 3).

### Ecological and evolutionary implications of introgression

While our analyses demonstrate introgression after a substantial period of little gene flow, many questions remain about its causes and adaptive consequences. One possible explanation for the increased uptake of *C. jejuni* DNA by *C. coli* lineages that are more common in agriculture (Table 2) is enhanced physical opportunity for genetic exchange associated with cocolonization of agricultural hosts by the two species. Little is known about the host range for nonagricultural *C. coli*, which include clades 2 and 3 and unintrogressed clade 1 isolates (Sheppard *et al*. 2010b), but it might be principally composed of reservoirs that are not colonized by *C. jejuni*. One challenge for a simple model in which recombination rates are regulated by physical proximity is the low rate with which agricultural *C. jejuni* have acquired *C. coli* DNA, although this might in part be explained by a higher frequency of *C. jejuni* in hosts in which they co-occur.

Given the absence of unintrogressed *C. coli* in food animals, it is possible to speculate that introgression may have provided key adaptations for proliferation in the agricultural niche. Introgression has led to *C. coli* clade 1 having the largest pan genome (Fig. S9, Supporting information) and to the import of several genes involved in the transport and metabolism of L-fucose (Table 1), which has been shown to be important in colonizing hosts (Muraoka & Zhang 2011). There are other examples of pathogenic lineages that are proposed to have arisen after a rapid burst of genome-wide introgression. These include *Salmonella Paratyphi* A and *Typhi* (Didelot *et al*. 2007) and *Vibrio vulnificus* (Bisharat *et al*. 2005). However, in *Campylobacter*, the genetic distance between hybridizing lineages is far greater and is comparable to that between *Escherichia coli* and *Salmonella* (Ochman & Groisman 1994) or a human and a marmoset (Peng *et al*. 2009).

Whatever its adaptive benefits, the interspecies recombination that has been observed in *C. coli* presents a challenge to some views of how bacterial evolution proceeds. It has been proposed that most changes in proteins occur by coevolution, with substitutions in one protein resulting in selection pressure for reciprocal changes in interacting partners (Fraser *et al*. 2002). Recombination between divergent species would be expected to disrupt large numbers of evolved interactions and, if the genetic distance was sufficiently great, would be likely to create hybrids, or ‘hopeful monsters’ (Mayr 1970), with little chance of evolutionary success. Here, there is an average of 40 protein coding differences between the two species.

To surmount the deleterious effects of disrupting numerous epistatic interactions, there would need to be substantial fitness advantages. Novel or extreme environments provide a setting within which hopeful monsters can be generated and proliferate because of the absence of well-adapted organisms (Rieseberg *et al*. 2003). There are many features that might make livestock such a habitat for bacteria that have evolved in wild hosts. Furthermore, hopeful bacterial monsters can repair some of the most harmful disruptions to interactions of adaptive genes by subsequent homologous recombination of their interaction partners. Sequencing of larger numbers of isolates will allow more detailed characterization of this ongoing adaptive process and further develop our understanding of bacterial gene networks.

## Data accessibility

Whole genome sequences (contigs assembled in VELVET) are deposited in the Dryad repository, doi:10.5061/dryad.215jd. DNA sequences are available from NCBI with the following accession numbers: ANGL00000000, ANGM00000000, ANGN00000000, ANGO00000000, ANGP00000000, ANGQ00000000, ANGR00000000, ANGS00000000, ANGT00000000, ANGU00000000, ANGV00000000, ANGW00000000, ANGX00000000, ANGY00000000, ANGZ00000000, ANHA00000000, ANHB00000000, ANHC00000000, ANHD00000000, ANHE00000000, ANHF00000000, ANHG00000000, ANHH00000000, ANHI00000000, ANHJ00000000, ANHK00000000.

## Supporting information

**Fig. S1** *Campylobacter* genome analysis pipeline.

**Fig. S2** Histograms of nucleotide divergence between *C. coli* clade 1 isolates (17 and 18) from the ST-828 (A) and ST-1150 (B) clonal complexes and *C. jejuni* isolates 14, 22, 26, 28, 29, 30.

**Fig. S3** Divergence between example *C. coli* clade 1 genomes from the ST-828 and ST-1150 clonal complexes and *C. jejuni*.

**Fig. S4** Frequency distribution of dn/ds ratios of 584 genes that have recombined between *C. jejuni* and *C. coli* clade 1 and 497 that have not.

**Fig. S5** Functional category of recombined genes.

**Fig. S6** Neighbor-joining trees of putative L-fucose pathway genes.

**Fig. S7** Ancestry of *C. coli* isolates from a published study.

**Fig. S8** Neighbour-joining trees for the 13 genes, also present in the analysis of Lefebure *et al*. (2010), with highest introgression into ST-828 complex - estimated using STRUCTURE.

**Fig. S9** *C. coli* and *C. jejuni* core and pan-genome content according to the number of sequenced genomes.

**Table S1** Isolate details.

**Table S2** Isolate genome details.

**Table S3** *C. jejuni* genes absent from unrecombined *C. coli*.

## References

[b1] Altschul SF, Gish W, Miller W, Myers EW, Lipman DJ (1990). Basic local alignment search tool. Journal of Molecular Biology.

[b2] Bisharat N, Cohen DI, Harding RM (2005). Hybrid *Vibrio vulnificus*. Emerging Infectious Diseases.

[b3] Cabello H, Torres A, Celis R (1997). Bacterial colonization of distal airways in healthy subjects and chronic lung disease: a bronchoscopic study. European Respiratory Journal.

[b4] Caro-Quintero A, Rodriguez-Castano GP, Konstantinidis KT (2009). Genomic insights into the convergence and pathogenicity factors of *Campylobacter jejuni* and *Campylobacter coli* species. Journal of Bacteriology.

[b5] Cohan FM (2002). Sexual isolation and speciation in bacteria. Genetica.

[b6] Cohan FM, Koeppel AF (2008). The origins of ecological diversity in prokaryotes. Current Biology.

[b7] Colles FM, Dingle KE, Cody AJ, Maiden MC (2008). Comparison of *Campylobacter* populations in wild geese with those in starlings and free-range poultry on the same farm. Applied and Environmental Microbiology.

[b8] Consortium(TCSaA) (2005). Initial sequence of the chimpanzee genome and comparison with the human genome. Nature.

[b9] Darling AC, Mau B, Blattner FR, Perna NT (2004). Mauve: multiple alignment of conserved genomic sequence with rearrangements. Genome Research.

[b10] Darling AE, Mau B, Perna NT (2010). progressiveMauve: multiple genome alignment with gene gain, loss and rearrangement. PLoS One.

[b11] Didelot X, Falush D (2007). Inference of bacterial microevolution using multilocus sequence data. Genetics.

[b12] Didelot X, Achtman M, Parkhill J, Thomson NR, Falush D (2007). A bimodal pattern of relatedness between the Salmonella Paratyphi A and Typhi genomes: convergence or divergence by homologous recombination?. Genome Research.

[b13] Didelot X, Darling A, Falush D (2009). Inferring genomic flux in bacteria. Genome Research.

[b14] Doolittle WF (2008). Microbial evolution: stalking the wild bacterial species. Current Biology.

[b15] Doolittle WF, Zhaxybayeva O (2009). On the origin of prokaryotic species. Genome Research.

[b16] Doyle LP (1948). The etiology of swine dysentrey. American Journal of Veterinary Research.

[b17] Edgar RC (2004). MUSCLE: multiple sequence alignment with high accuracy and high throughput. Nucleic Acids Research.

[b18] Escherich T (1886). Beitrage zur Kenntniss der Darmbacterien. III. Ueber das Vorkommen von Vibrionen im Darmcanal und den Stuhlgangen der Sauglinge. (Articles adding to the knowledge of intestinal bacteria. III. On the existence of vibrios in the intestines and feces of babies.). Munchener Med Wochenschrift.

[b19] Falush D (2009). Toward the use of genomics to study microevolutionary change in bacteria. PLoS Genetics.

[b20] Falush D, Stephens M, Pritchard JK (2003). Inference of population structure using multilocus genotype data: linked loci and correlated allele frequencies. Genetics.

[b21] Fouts DE, Mongodin EF, Mandrell RE (2005). Major structural differences and novel potential virulence mechanisms from the genomes of multiple campylobacter species. PLoS Biology.

[b22] Fraser HB, Hirsh AE, Steinmetz LM, Scharfe C, Feldman MW (2002). Evolutionary rate in the protein interaction network. Science.

[b23] Fraser C, Hanage WP, Spratt BG (2007). Recombination and the nature of bacterial speciation. Science.

[b24] Gundogdu O, Bentley SD, Holden MT (2007). Re-annotation and re-analysis of the *Campylobacter jejuni* NCTC11168 genome sequence. BMC Genomics.

[b25] Hartley-Tassell LE, Shewell LK, Day CJ (2010). Identification and characterization of the aspartate chemosensory receptor of *Campylobacter jejuni*. Molecular Microbiology.

[b26] Jolley K, Maiden M (2010). BIGSdb: scalable analysis of bacterial genome variation at the population level. BMC Bioinformatics.

[b27] Jolley KA, Bliss CM, Bennett JS (2012). Ribosomal multilocus sequence typing: universal characterization of bacteria from domain to strain. Microbiology.

[b28] Kumar S, Nei M, Dudley J, Tamura K (2008). MEGA: a biologist-centric software for evolutionary analysis of DNA and protein sequences. Brief Bioinformatics.

[b29] Lawrence JG (1999). Gene transfer, speciation, and the evolution of bacterial genomes. Current Opinion in Microbiology.

[b30] Lefebure T, Bitar PD, Suzuki H, Stanhope MJ (2010). Evolutionary dynamics of complete Campylobacter pan-genomes and the bacterial species concept. Genome Biology and Evolution.

[b31] Mayr E, Mayr E (1970). Multiplication of species. Populations, Species, and Evolution: An Abridgment of Animal Species and Evolution.

[b32] Muraoka WT, Zhang Q (2011). Phenotypic and genotypic evidence for L-fucose utilization by *Campylobacter jejuni*. Journal of Bacteriology.

[b33] Ochman H, Groisman EA (1994). The origin and evolution of species differences in *Escherichia coli* and *Salmonella typhimurium*. EXS.

[b34] Ochman H, Lawrence JG, Groisman EA (2000). Lateral gene transfer and the nature of bacterial innovation. Nature.

[b35] Parkhill J, Wren BW, Mungall K (2000). The genome sequence of the food-borne pathogen *Campylobacter jejuni* reveals hypervariable sequences. Nature.

[b36] Parkhill J, Dougan G, James KD (2001). Complete genome sequence of a multiple drug resistant *Salmonella enterica* serovar Typhi CT18. Nature.

[b37] Pearson BM, Gaskin DJ, Segers RP (2007). The complete genome sequence of *Campylobacter jejuni* strain 81116 (NCTC11828). Journal of Bacteriology.

[b38] Peng Z, Elango N, Wildman DE, Yi SV (2009). Primate phylogenomics: developing numerous nuclear non-coding, non-repetitive markers for ecological and phylogenetic applications and analysis of evolutionary rate variation. BMC Genomics.

[b39] Retchless AC, Lawrence JG (2010). Phylogenetic incongruence arising from fragmented speciation in enteric bacteria. Proceedings of the National Academy of Sciences of the United States of America.

[b40] Rieseberg LH, Raymond O, Rosenthal DM (2003). Major ecological transitions in wild sunflowers facilitated by hybridization. Science.

[b41] Rosef O, Gondrosen B, Kapperud G, Underdal B (1983). Isolation and characterization of *Campylobacter jejuni* and *Campylobacter coli* from domestic and wild mammals in Norway. Applied and Environmental Microbiology.

[b42] Sebald M, Veron M (1963). [Base DNA content and classification of vibrios]. Annales de l'Institut Pasteur.

[b43] Sheppard SK, McCarthy ND, Falush D, Maiden MC (2008). Convergence of *Campylobacter* species: implications for bacterial evolution. Science.

[b44] Sheppard SK, Dallas JF, Strachan NJ (2009). *Campylobacter* genotyping to determine the source of human infection. Clinical Infectious Diseases.

[b45] Sheppard SK, Colles F, Richardson J (2010a). Host association of *Campylobacter* genotypes transcends geographic variation. Applied and Environmental Microbiology.

[b46] Sheppard SK, Dallas JF, Wilson DJ (2010b). Evolution of an agriculture-associated disease causing *Campylobacter coli* clade: evidence from national surveillance data in Scotland. PLoS One.

[b47] Sheppard SK, Maiden MCJ, Falush D, Robinson DA, Falush D, Feil EJ (2010c). Population genetics of Campylobacter. Bacterial Population Genetics in Infectious Disease.

[b48] Sheppard SK, McCarthy ND, Jolley KA, Maiden MC (2011). Introgression in the genus Campylobacter: generation and spread of mosaic alleles. Microbiology.

[b49] Snipen L, Wassenaar T, Altermann E (2012). Analysis of evolutionary patterns of genes in *Campylobacter jejuni* and *C. coli*. Microbial Informatics and Experimentation.

[b50] Sopwith W, Birtles A, Matthews M (2008). Identification of potential environmentally adapted *Campylobacter jejuni* strain, United Kingdom. Emerging Infectious Diseases.

[b51] Stahl M, Friis LM, Nothaft H (2011). L-fucose utilization provides *Campylobacter jejuni* with a competitive advantage. Proceedings of the National Academy of Sciences of the United States of America.

[b52] Suerbaum S, Lohrengel M, Sonneveld A, Ruberg F, Kist M (2001). Allelic diversity and recombination in *Campylobacter jejuni*. Journal of Bacteriology.

[b53] Thakur S, Morrow WE, Funk JA, Bahnson PB, Gebreyes WA (2006). Molecular epidemiologic investigation of *Campylobacter coli* in swine production systems, using multilocus sequence typing. Applied and Environmental Microbiology.

[b54] Treangen TJ, Darling AE, Achaz G (2009). A novel heuristic for local multiple alignment of interspersed DNA repeats. IEEE/ACM Transactions on Computational Biology and Bioinformatics.

[b55] Velayudhan J, Kelly DJ (2002). Analysis of gluconeogenic and anaplerotic enzymes in *Campylobacter jejuni*: an essential role for phosphoenolpyruvate carboxykinase. Microbiology.

[b56] Veron M, Chatelain R (1973). Taxonomic study of the genus *Campylobacter* and designation of the neotype strain for the type species *Campylobacter fetus*. International Journal of Systematic Bacteriology.

[b57] Waldenstrom J, Broman T, Carlsson I (2002). Prevalence of *Campylobacter jejuni*
*Campylobacter lari*, and *Campylobacter coli* in different ecological guilds and taxa of migrating birds. Applied and Environmental Microbiology.

[b58] Waldenstrom J, On SL, Ottvall R, Hasselquist D, Olsen B (2007). Species diversity of campylobacteria in a wild bird community in Sweden. Journal of Applied Microbiology.

