# Supplementary figures and images for: Progressive genome-wide introgression in agricultural Campylobacter coli
Source: Mol Ecol. 2012 Dec 20;22(4):1051–64. doi: 10.1111/mec.12162 (PMC3749442; doi:10.1111/mec.12162)

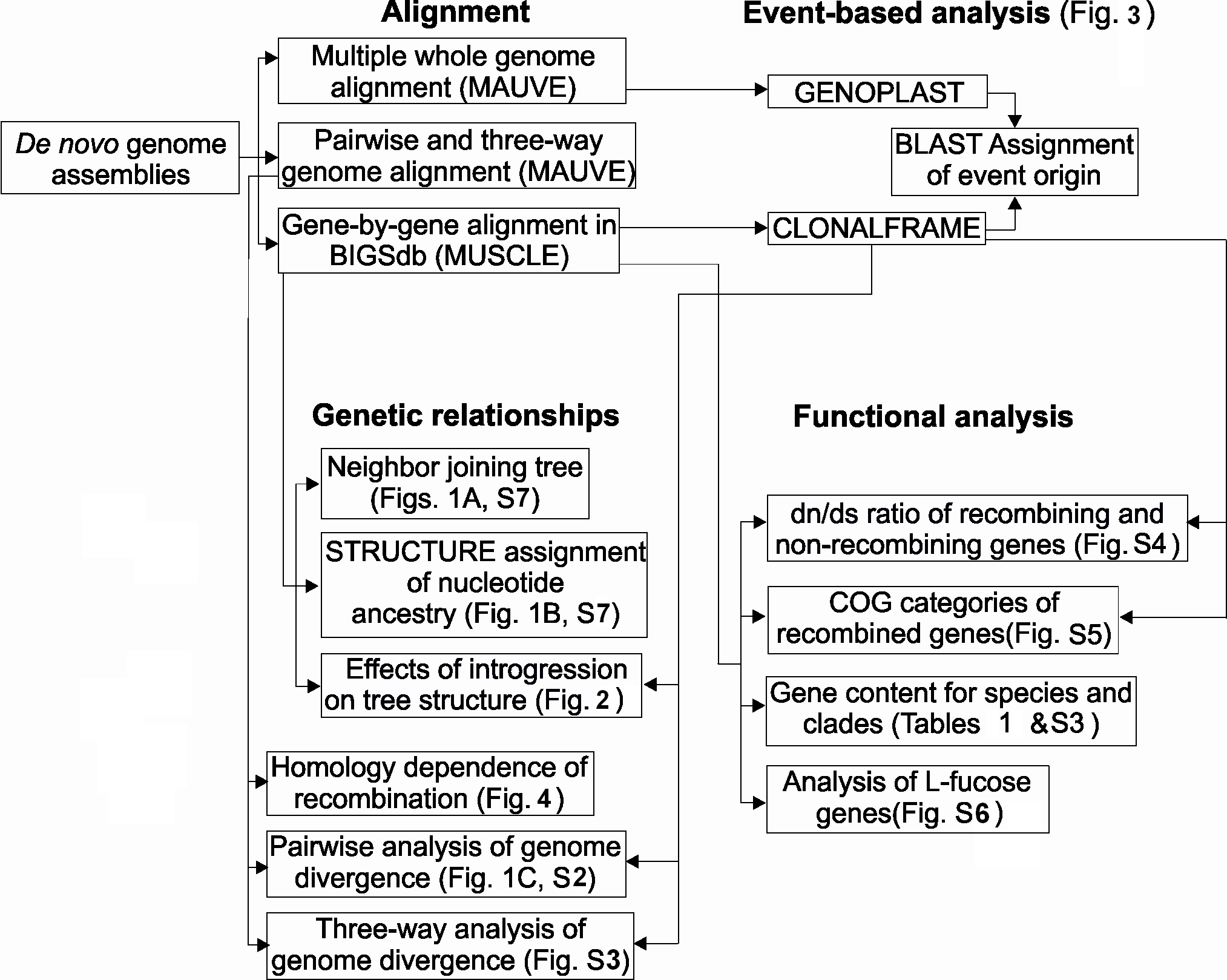

Supplement: Supplementary file 1 [file mec0022-1051-SD1.tif]

*C. jejuni* isolate

14  
[Environmental isolate]

22

26

28

29

30

number of sites (Kb)

**A**

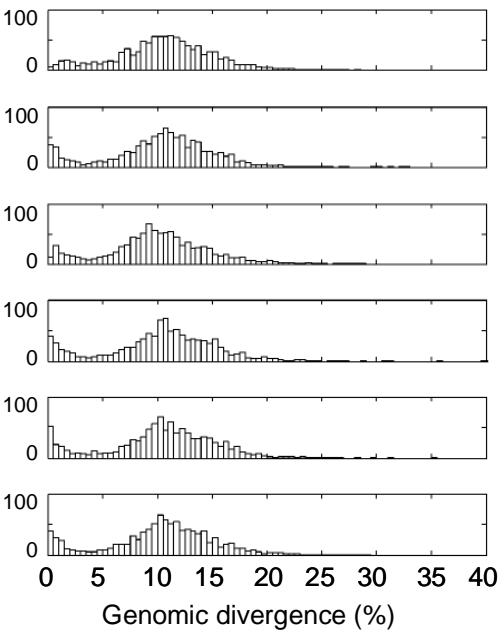

**B**

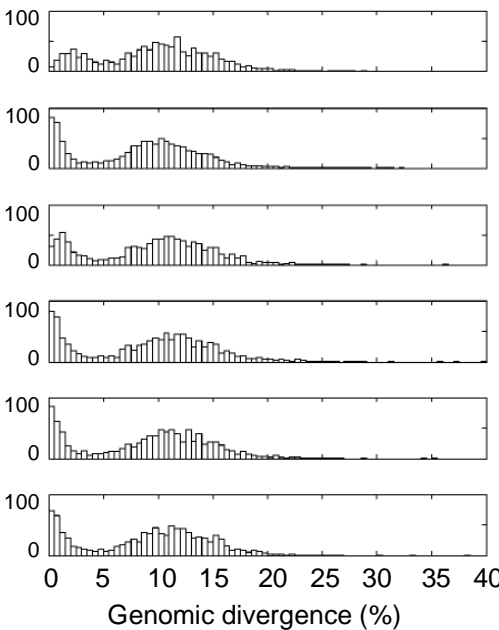

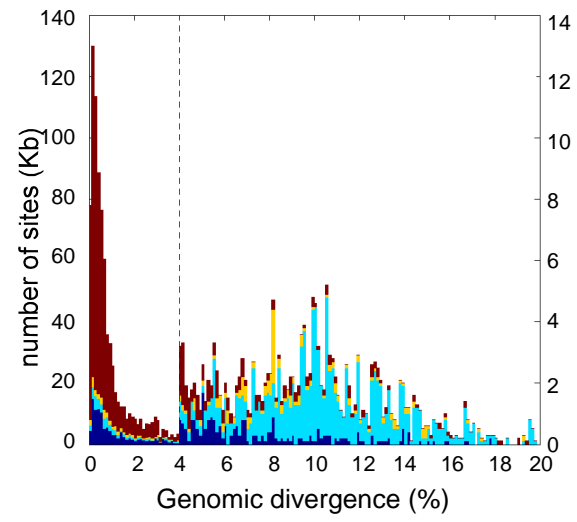

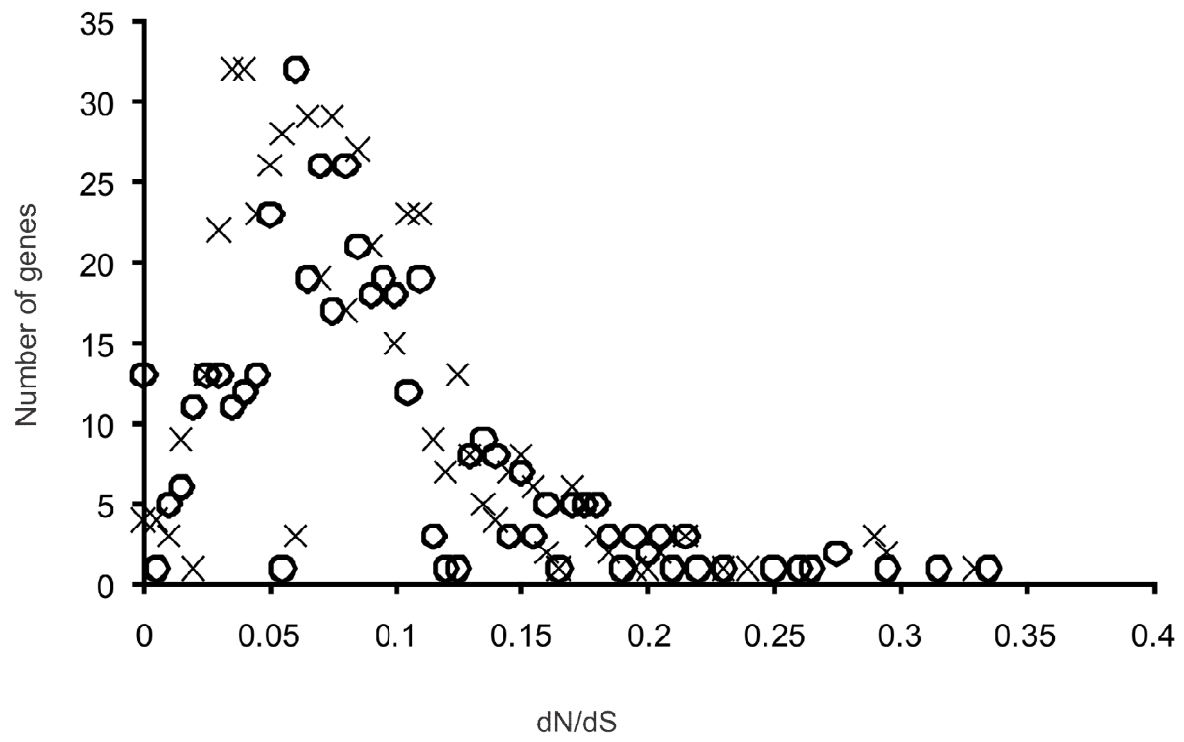

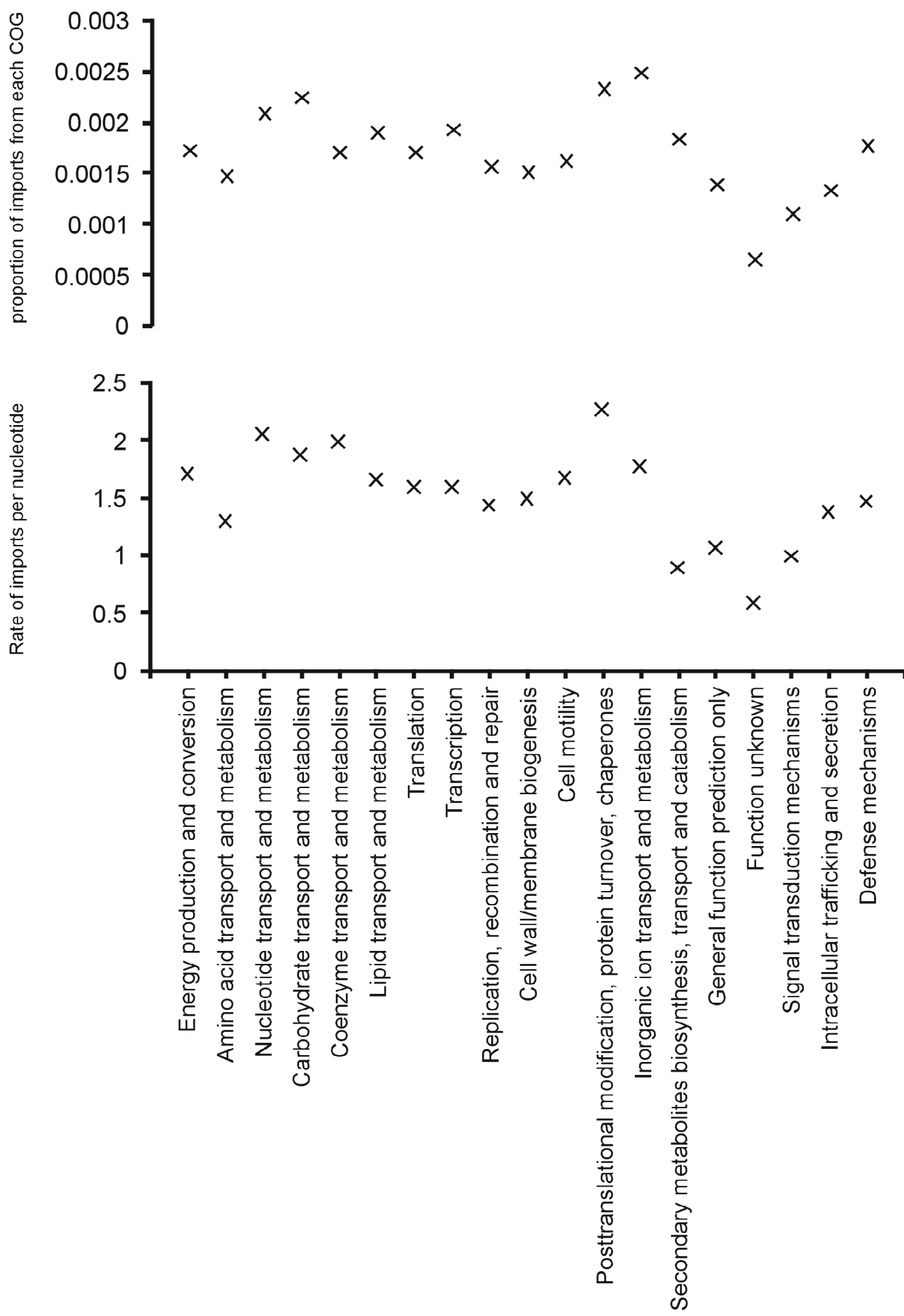

Cj0480

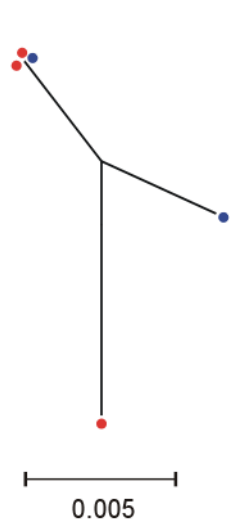

Cj0481

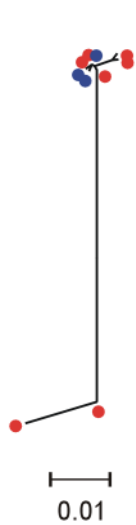

uxaA

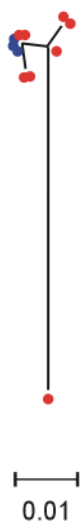

Cj0484

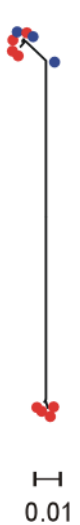

Cj0485

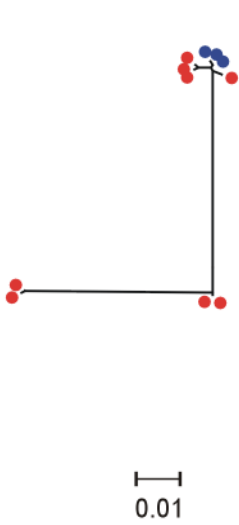

Cj0486

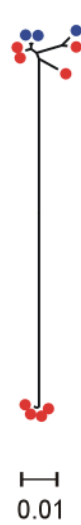



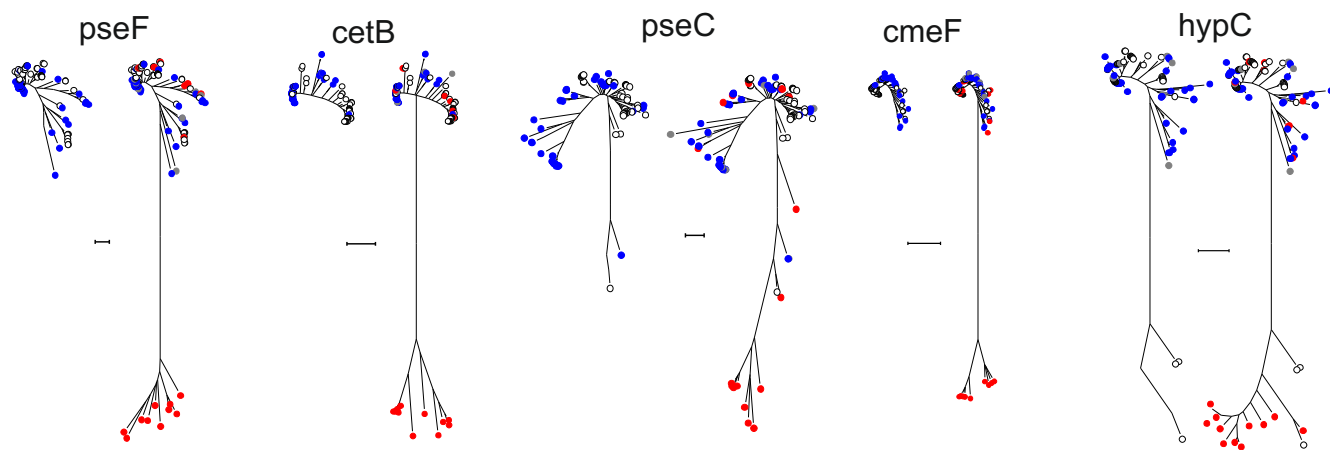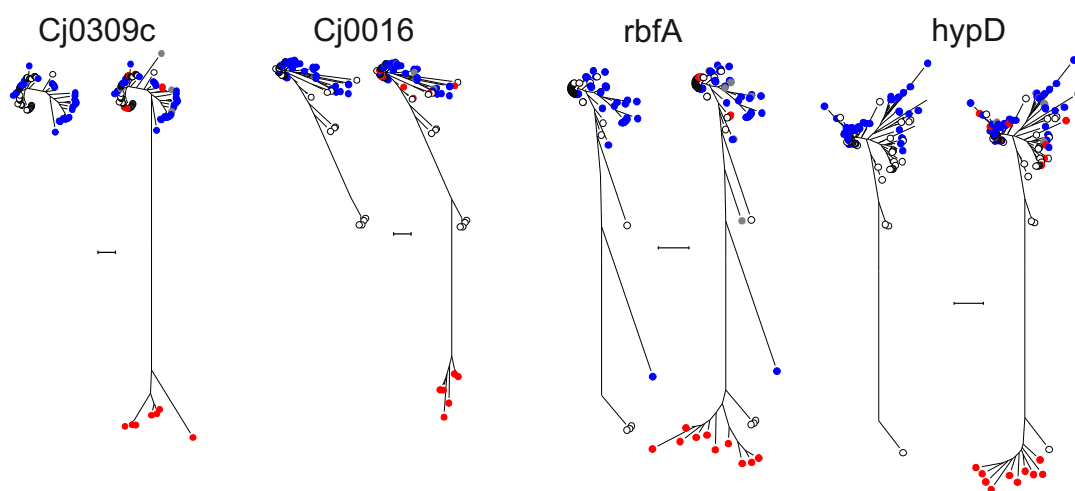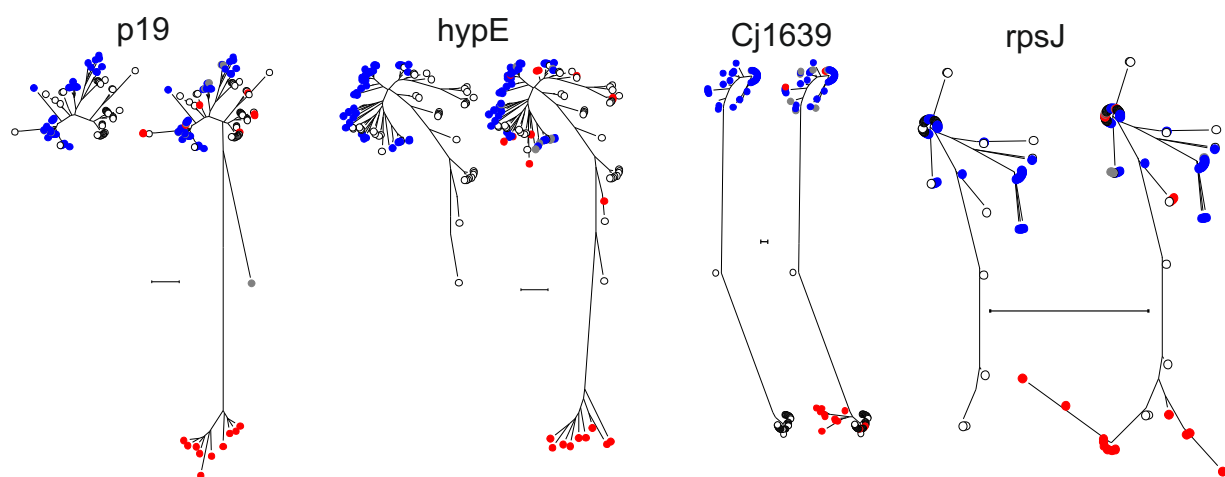

Supplement: Supplementary file 2 [file mec0022-1051-SD2.pdf]
