# Supplementary material for: Progressive genome-wide introgression in agricultural Campylobacter coli
Source: Mol Ecol. 2012 Dec 20;22(4):1051–64. doi: 10.1111/mec.12162 (PMC3749442; doi:10.1111/mec.12162)
Supplement: Supplementary file 3 [file mec0022-1051-SD3.docx]

**Table S1**. Isolate details.

| Species/clade | Isolate | Alias | Source | Country | Date of isolation | ST^1^ | Clonal complex^2^ |
| --- | --- | --- | --- | --- | --- | --- | --- |
| *C. jejuni* | C8 | 4 | Chicken meat | UK | 2005 | 45 | 45 |
| *C. jejuni* | 7802A | 13 | Cattle faeces | UK | 2006 | 61 | 61 |
| *C. jejuni* | W260a | 14 | Environmental water | NZ | 2001 | 2381 | - |
| *C. jejuni* | 48321 | 22 | Chicken meat | UK | 2001 |  |  |
| *C. jejuni* | NCTC11828, 81116 (NC 009839) | 26 | Clinical | UK | 2007 | 267 | 283 |
| *C. jejuni* | 81-176(NC 008787) | 27 | Clinical | USA | 2007 | 604 | 42 |
| *C. jejuni* | RM1221 (NC 003912) | 28 | Chicken meat | USA | 2002 | 354 | 354 |
| *C. jejuni* | NCTC11168 (NC 002163) | 29 | Clinical | UK | 2000 | 43 | 21 |
| *C. jejuni* | 7487 | 30 | Clinical | UK | 2006 | 1044 | 658 |
| *C. coli* – clade 1 | F79015 | 2 | Clinical | UK | 2003 | 867 | 828 |
| *C. coli* – clade 1 | 8993 | 5 | Pig faeces | UK | 2006 | 2696 | 828 |
| *C. coli* – clade 1 | PW1 | 15 | Pig faeces | UK | 2004 | 886 | 828 |
| *C. coli* – clade 1 | Duck269, 8840 | 16 | Duck faeces | UK | 2007 | 1771 | - |
| *C. coli* – clade 1*­* | C138 | 17 | Chicken meat | UK | 2005 | 2588 | 828 |
| *C. coli* – clade 1 | C28B51 | 18 | Chicken faeces | UK | 2004 | 3667 | 1150 |
| *C. coli* – clade 1 | 182 | 19 | Clinical | UK | 2006 | 3129 | 828 |
| *C. coli* – clade 1 | C4B19 | 20 | Chicken faeces | UK | 2003 | 1487 | 1150 |
| *C. coli* – clade 1 | C4B30 | 21 | Chicken faeces | UK | 2003 | 1090 | 828 |
| *C. coli* – clade 1 | Duck323, 8866 | 23 | Duck faeces | UK | 2007 | 3311 | - |
| *C. coli* – clade 1 | BB2617 | 24 | Chicken faeces | UK | 2003 | 828 | 828 |
| *C. coli* – clade 1 | 911 | 25 | Clinical | UK | 2005 | 3136 | 1150 |
| *C. coli* – clade 2 | 8808 | 1 | Duck faeces | UK | 2007 | 3304 | - |
| *C. coli* – clade 2 | 2544 | 10 | Clinical | UK | 2006 | 2326 | - |
| *C. coli* – clade 2 | 6873 | 11 | Duck faeces | UK | 2006 | 2016 | - |
| *C. coli* – clade 2 | dfvf1656 | 12 | Chicken meat | DK | 2001 | 1572 | - |
| *C. coli* – clade 3 | FSA05.280042 | 3 | Environmental waters | UK | 2005 | 1992 | - |
| *C. coli* – clade 3 | 8096 | 6 | Chicken meat | UK | 2006 | 2681 | - |
| *C. coli* – clade 3 | dfvf1912 | 7 | Chicken meat | DK | 2002 | 1576 | - |
| *C. coli* – clade 3 | 4944 | 8 | Clinical | USA | 2001 | 1670 | - |
| *C. coli* – clade 3 | RM4931 | 9 | Clinical | USA | 2000 | 1643 | - |

^1^Sequence type (ST) was derived from the allelic profile of 7 housekeeping genes by multilocus sequence typing (MLST) and confirmed by whole genome sequencing.

^2^Clonal complexes are defined as including any ST that matches a previously defined central genotype (http://pubmlst.org/campylobacter/) at three or more loci.
