# Supplementary material for: Progressive genome-wide introgression in agricultural Campylobacter coli
Source: Mol Ecol. 2012 Dec 20;22(4):1051–64. doi: 10.1111/mec.12162 (PMC3749442; doi:10.1111/mec.12162)
Supplement: Supplementary file 4 [file mec0022-1051-SD4.docx]

**Table S2.** Isolate genome details.

| Isolate | Alias | Total sequence length (bp) | Contigs^1^ | | |
| --- | --- | --- | --- | --- | --- |
|  |  |  | number | Max. length | Mean length |
| C8 | 4 | 1596969 | 108 | 86408 | 14787 |
| 7802A | 13 | 1551351 | 86 | 82676 | 18039 |
| W260a | 14 | 1543877 | 324 | 48068 | 4766 |
| 48321 | 22 | 1573736 | 169 | 46541 | 9313 |
| 7487 | 30 | 1613621 | 465 | 33617 | 3471 |
| F79015 | 2 | 1678444 | 184 | 55385 | 9122 |
| 8993 | 5 | 1589408 | 255 | 51401 | 6233 |
| PW1 | 15 | 1594196 | 216 | 37781 | 7381 |
| Duck269, 8840 | 16 | 813032 | 866 | 5536 | 939 |
| C138 | 17 | 1633576 | 79 | 162141 | 20679 |
| C28B51 | 18 | 1618028 | 206 | 40758 | 7855 |
| 182 | 19 | 1678945 | 276 | 62355 | 6084 |
| C4B19 | 20 | 1505418 | 691 | 14953 | 2179 |
| C4B30 | 21 | 1566888 | 374 | 27355 | 4190 |
| Duck323, 8866 | 23 | 1431051 | 446 | 20545 | 3209 |
| BB2617 | 24 | 1570976 | 532 | 18298 | 2953 |
| 911 | 25 | 1657253 | 261 | 38871 | 6350 |
| 8808 | 1 | 1752685 | 167 | 82635 | 10496 |
| 2544 | 10 | 1660889 | 191 | 54052 | 8696 |
| 6873 | 11 | 1606337 | 312 | 27178 | 5149 |
| dfvf1656 | 12 | 1554713 | 282 | 37772 | 5514 |
| FSA05.280042 | 3 | 1624910 | 125 | 139262 | 13000 |
| 8096 | 6 | 1542097 | 65 | 118285 | 23725 |
| dfvf1912 | 7 | 1521545 | 197 | 43967 | 7724 |
| 4944 | 8 | 1547646 | 281 | 26227 | 5508 |
| RM4931 | 9 | 1500025 | 217 | 37397 | 6913 |

^1^Contiguous sequence assemblies >500bp.
