# Supplementary material for: Progressive genome-wide introgression in agricultural Campylobacter coli
Source: Mol Ecol. 2012 Dec 20;22(4):1051–64. doi: 10.1111/mec.12162 (PMC3749442; doi:10.1111/mec.12162)
Supplement: Supplementary file 5 [file mec0022-1051-SD5.docx]

**Table S3.** *C. jejuni* genes absent from unrecombined *C. coli*.

| Gene | Product |
| --- | --- |
| **RNA metabolism** | |
| Cj0153c | rRNA methylase |
| Cj0327 | Endoribonuclease |
| Cj1280c | ribosomal pseudouridine synthase |
| gatC | Glu tRNA syntase |
| ksgA | 16S RNA demethylase |
| leuS | Leu tRNA synthase |
| truB | tRNA pseudouridine synthase |
| **DNA replication/repair** | |
| Cj0011c | DNA binding protein |
| Cj1101 | Helicase |
| Cj0481c | Helicase |
| Cj1482c | unknown, but may be related to 1481 function |
| Cj1669c | DNA ligase |
| Ogt | DNA methyltransferase |
| recR | Recombination protein |
| **Oxidative stress** | |
| MsrA | methionine sulphoxide reductase. Oxidative stress resistance |
| Cj0019c | MCP co-transcribed with Cj0020 (Cyt c peroxidase) |
| Cj0020c | Periplasmic Cyt c peroxidise |
| **Cell wall modification/biosynthesis** | |
| Alr | Alanine racemase |
| amiA | cell wall amidase |
| Cj0085c | Ala or Glu racemase |
| **TonB-dependent/Outer membrane (OM) receptors /OM function** | |
| Cj1086c | TonB dependent receptor, potentially in OM |
| htrB | Lipid A biosynthesis |
| tonB2 | TonB paralogue (CM to OM energy transduction) |
| ctsE | Type II secretion system |
| Cj0975 | OM protein |
| exbD2 | TonB complex protein (CM to OM energy transduction) |
| **Periplasmic cytochrome c biogenesis/Dsb system** | |
| Cj1013c | Cyt c biogenesis protein |
| Cj1207c | perilasmic thioredoxin like protein, potentially involved in cyt c biogenesis |
| dsbB | periplasmic disulphide bond oxidation system |
| Cj0158c | Heam containing lipoprotein. Possible cyt c or involved in cyt c biogenesis |
| Cj0874c | Di-heam cytochrome c |

**Table S3 continued.** *C. jejuni* genes absent from unrecombined *C. coli*.

| Gene | Product |
| --- | --- |
| **Solute transporters** | |
| Cj0339 | Major facilitator Superfamily solute transporter |
| Cj1241 | Major facilitator Superfamily solute transporter |
| Cj1538c | ATP binding protein of a Tungstate ABC transporter |
| glnP | Possible glutamine periplasmic binding protein of ABC system |
| livH | Branch chain amino acid ABC transporter permease protein |
| livM | Branch chain amino acid ABC transporter permease protein |
| sdaC | Serine transporter, HAAP family |
| chuD | Heam uptake protein |
| Cj0143c | ZnuA, periplasmic zinc binding protein of ABC system |
| Cj0186c | TerC related membrane protein |
| Cj0555 | MatC like possible malonate transporter |
| Cj0155c | cation transporting ATPase |
| Cj1161c | cation transporting ATPase |
| kdpB | Possible K+ transporting ATPase component |
| kdpD | Possible pseudogene K+ transport sensor kinase with Kinase domain absent |
| **Amino acid biosynthesis** | |
| thrB | Homoserine kinase, threonine biosynthesis |
| trpF | Phosphoribosyl anthranilate isomerise |
| **DNA metabolism/biosynthesis** | |
| apt | Adenenine phosphoribosyl transferase |
| Cj0340 | Nucleoside hydrolase |
| Cj1237c | putative phosphastase |
| pyrC2 | Dihydroorotase (Pyrimidine biosynthesis) |
| **Cofactor biosynthesis** | |
| bioD | Biotin synthesis |
| folC | Dihydrofolate synthase (Folate synthesis) |
| Cj0154c | tetrapyrrole methylase (heam biosynthesis) |
| **Membrane/lipid biosynthesis** | |
| aas | Acyl-transferase/acyl carrier protein |
| acpS | Acyl carrier protein synthase |
| Cj1115c | Phosphatidylserine decarboxylase |

**Table S4 continued.** *C. jejuni* genes absent from unrecombined *C. coli*.

| **Gene** | **Product** | |
| --- | --- | --- |
| **Miscellaneous/unknown** | | |
| Cj0090 | | Lipoprotein |
| Cj0463 | | Zn protease |
| Cj0495 | | Methyltransferase |
| Cj0504c | | NAD binding oxidoreductase |
| Cj0605 | | Amidohydroloase |
| Cj0620 | | Zn protease |
| Cj0796c | | Hydrolase |
| Cj0809c | | Hydrolase |
| Cj0829c | | CoA binding protein |
| Cj1589 | | Zn dependent hydrolase |
| Cj1649 | | Lipoprotein |
| Cj1666c | | periplasmic protein |
| pglB | | glycosylation protein |
| Cj0089 | | Lipoprotein |
| motB | | flagellar motor protein |
| Cj0230c | | Transferase |
| Cj0717 | | ArsC, arsenate oxidase |
| Cj0203 | | Unknown |
| Cj0873c | | Unknown |
| Cj0128c | | Inositol monophosphatase |
| rpiB | | ribose 5-phosphate isomerase (pentose phosphate pathway) |
| Cj0415 | | Gluconate dehydrogenase subunit |
| tlyA | | potential haemolysin |
| Cj0101 | | ParB |
| Cj0135 | | Unknown |
| ftsK | | cell division |
| Cj0248 | | Unknown |
| Cj1002c | | phosphoglycerate mutase |
| Cj1506c | | Unknown |
