# Supplementary material for: Progressive genome-wide introgression in agricultural Campylobacter coli
Source: Mol Ecol. 2012 Dec 20;22(4):1051–64. doi: 10.1111/mec.12162 (PMC3749442; doi:10.1111/mec.12162)
Supplement: Supplementary file 6 [file mec0022-1051-SD6.doc]

**Supplementary Methods**

***Isolate culturing and genome sequencing.***

Isolates were subcultured on Columbia Blood Agar (CBA) and plates were incubated for 48 hours in a Microaerophilic Workstation under microaerobic conditions (5% CO_2_, 5% O_2_, 3% H_2_ and 87% N_2_) at 42°C. Cell suspensions of each culture made in 125 μl phosphate-buffered saline or in water (Sigma Aldrich, UK) in a 0.2 ml PCR tube. Genomic DNA extraction was carried out using the QIAamp® DNA Mini Kit (QIAGEN GMBH, Hilden, Germany). The DNA was resuspended in 100-200 μl of the elution buffer supplied, and stored at – 20 °C.

For isolates 1-3 genomic DNA was sheared and 12kb paired end libraries were sequenced on a single Roche GS-FLX at 454 Life Sciences (USA). *de novo* assembly was carried out using Newbler, with individual base calls filtered to exclude nucleotides that were within 3 base pairs of an alignment gap, of low quality score (below 10), and contained greater than 30% mismatches within a 7 base pair window. This gave data files with between 125 and 184 contiguous sequences (contigs) of length > 500bp (Table S2).

Isolates 4-25 and 30 were sequenced using an Illumina Genome Analyzer using a multiplex sequencing approach with 12 separately tagged libraries sequenced simultaneously in two lanes of an 8 channel GAII flow cell. Libraries were created using the standard Illumina Indexing protocol. Briefly, (i) 2µg genomic DNA was fragmented by acoustic shearing to enrich for 200 bp fragments using a Covaris E210 and cleaned and end-repaired. (ii) A-tailing was carried out and (iii) adapters were ligated. (iv) To introduce specific tag sequences between the sequencing and flowcell binding sites of the Illumina adapter, an overlap extension PCR was carried out using the Illumina 3 primer set. Each of these steps was followed by a DNA cleanup using a 1:1 ratio of Ampure paramagnetic beads (Beckman Coulter, Inc., USA) to remove DNA <150 bp. Finally DNA quantification was carried out by qPCR followed by sequencing. The average overall output was 80 Mbp per isolate. High coverage short reads (25–50 bp) were assembled *de novo* using Velvet software to produce contigs of up to 162kb (Table S2).

***Ribosomal protein locus trees.***

In addition to determining the clonal genealogy and the origin of recombination events information from the clonalframe analysis was used to investigate the impact of homologous recombination with *C. jejuni* on sequence divergence in *C. coli* clade 1. This used sequence variation at 51 ribosomal protein (*rps*) subunit loci, excluding two loci that were apparently missing from the genomes, or were only partial coding sequences that were situated at the ends of contigs. Of these 51 genes, 16 (*rplB,C,D,E,I,P,W,X; rpmA; rpsB,D,G,H,K,L,M*) were identified by clonalframe to have been involved in homologous recombination with *C. jejuni* in at least one *C. coli* clade 1 isolate. A total of 35 *rps* genes (*rplA,F,J,K,L,M,N,O,Q,R,S,T,U,V*; *rpmB,C,E,F,G,H,I,J; rpsA,C,E,F,I,J,N,P,Q,R,S,T,U*) were not involved in recombination with *C. jejuni.* Gene orthologs for recombining (16 loci, 7650bp) and non-recombining (35 loci, 12828bp) loci were aligned on a gene-by-gene basis using muscle [49] and then concatenated into contiguous sequence for each isolate genome including gaps for missing nucleotides. The trees were inferred using the Maximum Likelihood method based on the Tamura-Nei model in MEGA5. The tree with the highest log likelihood (-22157.9550) is shown.
