# Supplementary material for: Progressive genome-wide introgression in agricultural Campylobacter coli
Source: Mol Ecol. 2012 Dec 20;22(4):1051–64. doi: 10.1111/mec.12162 (PMC3749442; doi:10.1111/mec.12162)
Supplement: Supplementary file 7 [file mec0022-1051-SD7.docx]

Fig. S1. *Campylobacter* genome analysis pipeline.

Fig. S2. Histograms of nucleotide divergence between *C. coli* clade 1 isolates (17 and 18) from the ST-828 (A) and ST-1150 (B) clonal complexes and *C. jejuni* isolates 14, 22, 26, 28, 29, 30. Pairs of isolates were aligned and analysed separately to determine the divergence levels at all sites along the genomes. Bimodal distributions of genomic divergence between *C. jejuni* and *C. coli* show a first peak at <5% indicative of recent introgression and a second peak at approximately 12%. Introgressed *C. coli* DNA is less similar to that from the environmental *C. jejuni.*

Fig. S3. Divergence between example *C. coli* clade 1 genomes from the ST-828 and ST-1150 clonal complexes and *C. jejuni*. Paired genomes were aligned and genome positions were colour-coded according to the level of nucleotide divergence from *C. jejuni* as follows: both genomes <6% divergent from *C. jejuni* (dark blue); ST-1150 <6 % divergent from *C. jejuni* and ST-828 >6% divergent from *C. jejuni* (light blue); ST-828 <6 % divergent from *C. jejuni* and ST-1150 >6% divergent from *C. jejuni* (yellow); both genomes >6% divergent from *C. jejuni* (brown). The dashed line denotes a switch to the second Y-axis scale. In regions where lineages from the ST-828 and ST-1150 complexes are divergent, this is largely the result of introgressed DNA from *C. jejuni.*

Fig. S4. Frequency distribution of dn/ds ratios of 584 genes that have recombined between *C. jejuni* and *C. coli* clade 1 and 497 that have not*.* The sample mean for recombined (x) and non-recombined (o) genes were 0.121 and 0.127 respectively and there was no significant difference between the distributions.

Fig. S5. Functional category of recombined genes. The COG category of genes involved in homologous recombination between *C. jejuni* and *C. coli* clade 1 is listed on the X-axis. The Y-axis shows the proportion of genes from each COG category involved in recombination and the rate of import per nucleotide. There is no significant difference between the COG groups.

Fig. S6. Neighbor-joining trees of putative L-fucose pathway genes. Genes from *C. jejuni* genomes are shown in blue and those from *C. coli* in red. The scale bars beneath the trees for each allele represent genetic distance. Distinct genotypes among the *C. jejuni*-like sequences from *C. coli* suggest multiple introgression events.

Fig. S7. Ancestry of *C. coli* isolates from a published study. (A) Neighbor-joining tree of 30 *C. jejuni* and *C. coli* genomes from this study (solid circles) and 40 *C. coli* genomes from Lefebure *et al.* [20] (open circles) that are available on the NCBI sequence read archive database (SRP001790 and SRA010929). Isolates belonging to *C. jejuni* are shown in blue and those belonging to *C. coli* clade 1 are indicated in red, clade 2 in yellow, and clade 3 in green. All the Lefebure *et al.* isolates are from *C. coli* clade one, 39 from the ST-828 complex and 1 from the ST-1150 complex. The scale bar represents a genetic distance of 0.01. (B) Percentage of *C. coli* genomes originating in *C. jejuni* inferred using structure assuming 2 populations. Lefebure *et al.* isolates (white bars) and isolates from this study (black bars) are ordered according to the amount of introgression. The isolate marked * is an unintrogressed *C. coli* clade 1.

Fig. S8. Neighbour-joining trees for the 13 genes, also present in the analysis of Lefebure *et al.* (2010), with highest introgression into ST-828 complex - estimated using STRUCTURE. For each gene, two trees are shown, the first is based on the 83 genomes from Lefebure *et al.* [20] that are available on the NCBI sequence read archive database and the second additionally includes the 30 isolates from this study. *C. jejuni* are indicated by blue (lefebure *et al.*) and grey (this study) circles, *C. coli* from Lefebure *et al.* by white circles and *C. coli* from this study are in red. The scale bar for each gene represents a genetic distance of 0.01.

Fig. S9. *C. coli* and *C. jejuni* core and pan-genome content according to the number of sequenced genomes. Conserved orthologous sequence (A) and total amount of non-orthologous sequence (B) for a given number of genomes analysed for *C. coli* clade 1 (red), *C. coli* clade 2 (yellow), *C. coli* clade 3 (green), *C. coli* clades 1-3 (grey), *C. jejuni* (blue) and all of these groups combined (black). Mean values were calculated and trend lines are broken as they extend beyond the total number of genomes for each group. At 30 sequenced genomes, the core-genome had 958 Kb of orthologous sequence - 5% of the pan-genome for the equivalent sample group.
